# *In vitro* activity of salinomycin and monensin derivatives against *Trypanosoma brucei*

**DOI:** 10.1186/s13071-016-1698-8

**Published:** 2016-07-25

**Authors:** Dietmar Steverding, Michał Antoszczak, Adam Huczyński

**Affiliations:** 1 Bob Champion Research & Education Building, Norwich Medical School, University of East Anglia, Norwich, UK; 2 Faculty of Chemistry, Adam Mickiewicz University, Poznań, Poland

**Keywords:** *Trypanosoma brucei*, Salinomycin derivatives, Monensin derivatives, African trypanosomaisis, Drug screening

## Abstract

**Background:**

African trypanosomes are the causative agents of sleeping sickness in humans and nagana disease in livestock animals. As the few drugs available for treatment of the diseases have limited efficacy and produce adverse reactions, new and better tolerated therapies are required. Polyether ionophores have been shown to display anti-cancer, anti-microbial and anti-parasitic activity. In this study, derivatives of the polyether ionophores, salinomycin and monensin were tested for their *in vitro* activity against bloodstream forms of *Trypanosoma brucei* and human HL-60 cells.

**Results:**

Most polyether ionophore derivatives were less trypanocidal than their corresponding parent compounds. However, two salinomycin derivatives (salinomycin n-butyl amide and salinomycin 2,2,2-trifluoroethyl ester) were identified that showed increased anti-trypanosomal activity with 50 % growth inhibition values in the mid nanomolar range and minimum inhibitory concentrations of below 1 μM similar to suramin, a drug used in the treatment of sleeping sickness. In contrast, human HL-60 cells were considerably less sensitive towards all polyether ionophore derivatives. The cytotoxic to trypanocidal activity ratio (selectivity) of the two promising compounds was greater than 250.

**Conclusions:**

The data indicate that polyether ionophore derivatives are interesting lead compounds for rational anti-trypanosomal drug development.

## Background

African trypanosomiasis is an infectious parasitic disease of humans and animals of similar aetiology and epidemiology. The causative agents of the disease are flagellated protozoans of the genus *Trypanosoma*. The parasites are transmitted by the bite of infected tsetse flies (*Glossina* sp.) and live and multiply in the blood and tissue fluids of their mammalian host. The distribution of trypanosomiasis in Africa corresponds to the range of tsetse flies and comprises an area of 8 million km^2^ between 14°N and 20°S latitude [1]. In this so-called tsetse belt, millions of people and cattle are at risk of contracting the disease [2, 3]. Throughout history, African trypanosomiasis has severely repressed the economic and cultural development of central Africa [4].

For treatment of African trypanosomiasis only a handful drugs are available. All the drugs are outdated, require parenteral administration, induce significant toxic side effects, have limited efficacy and are being increasingly subject to drug resistance [5–7]. Thus, there is an urgent need for the development of new, more effective and safer treatments for African trypanosomiasis.

In recent years, polyether ionophores have received attention as promising anti-cancer candidate drugs [8]. However, compounds displaying anti-cancer activity often also exhibit trypanocidal activity [9]. Recently it has been shown that the ionophore salinomycin inhibits the growth of bloodstream forms of *T. brucei in vitro* at sub-micromolar concentration [10]. Although salinomycin was shown to be less toxic to human cells, its selectivity (cytotoxic/trypanocidal ratio) was in a moderate range (< 100) [10]. Therefore, we were interested whether chemical modification of polyether ionophores, such as salinomycin and monensin, could lead to compounds with improved trypanocidal activity and better selectivity.

## Methods

### Compounds

**Fig. 1:**
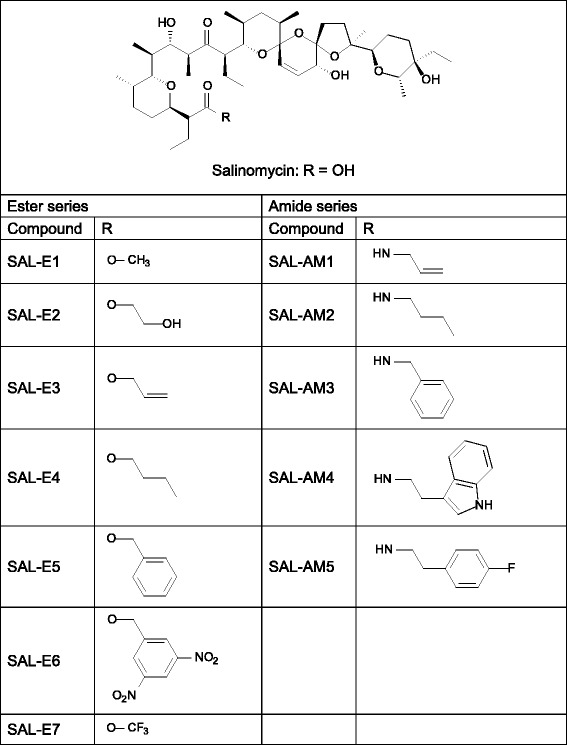
Structure of salinomycin and its derivatives studied in this work

**Fig. 2:**
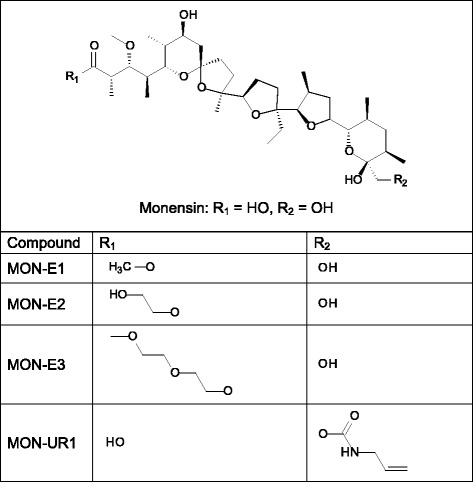
Structure of monensin and its derivatives studied in this work

The synthesis of the twelve salinomycin derivatives (Fig. 1) and the four monensin derivatives (Fig. 2) investigated in this study is described elsewhere [11–17].

### Cell cultures

Bloodstream forms of *T. brucei* (clone 427-221a) [18] and human myeloid leukaemia HL-60 cells [19] were grown in Baltz medium [20] and RPMI medium [21], respectively. Both culture media were supplemented with 16.7 % (v/v) heat-inactivated foetal calf serum. All cultures were maintained in a humidified atmosphere containing 5 % CO_2_ at 37 °C.

### Toxicity assays

Cells were seeded in 96-well plates in a final volume of 200 μl of their respective culture medium containing 10-fold serial dilutions of ionophore derivatives (10^-4^ to 10^-10^ M) and 1 % DMSO. Wells containing medium and 1 % DMSO served as controls. The initial cell densities were 1 × 10^4^/ml for *T. brucei* and 1 × 10^5^/ml for HL-60 cells. After 24 h incubation at 37 °C in a humidified atmosphere containing 5 % CO_2_, 20 μl of a 0.44 mM resazurin solution prepared in PBS was added and the cells were incubated for a further 48 h so that the total incubation time was 72 h. Thereafter, the plates were read on a microplate reader using a test wavelength of 570 nm and a reference wavelength of 630 nm. The 50 % growth inhibition (GI_50_) value, i.e. the concentration of a compound necessary to reduce the growth rate of cells by 50 % compared to the control was determined by linear interpolation according to the method described in [22]. The minimum inhibitory concentration (MIC) values, i.e. the concentration of the drug at which all trypanosome and human cells were killed, was determined microscopically. Each compound was independently tested three times.

### Measurement of changes in cell volume

Change in cell volume was determined by light scattering as previously described [10]. In brief, bloodstream forms of *T. brucei* were seeded at a density of 5 × 10^7^ cells/ml in 96-well plates in a final volume of 200 μl culture medium containing 100 μM of salinomycin or the salonimycin derivatives SAL-E7 or SAL-AM2 and 1 % DMSO. Absorbance of the cultures was measured at 490 nm every 15 min. A decrease in absorbance corresponded to an increase in cell volume. The experiment was repeated three times.

## Results and discussion

It has been shown that chemical modification of polyether ionophore not only can increase their anti-cancer and anti-bacterial activity but also can reduce their general cytotoxicity [23]. In addition, salinomycin and monensin with modified carboxyl groups (esters or amides) transport cations via an electrogenic or biomimetic mechanism while the unmodified parent ionophores carry cations across membranes always by an electroneutral mechanism [6]. This change in ionophoretic properties can lead to compounds with better biological activities. Most salinomycin and all monensin derivatives tested in this study were found to be less trypanocidal than their parent compounds with MIC values of 10 μM and GI_50_ values of around 3 μM (Table 1). Only the salinomycin derivatives SAL-E7 (2,2,2-trifluoroethyl ester) and SAL-AM2 (*n*-butyl amide) displayed increased trypanocidal activity with MIC values ranging between 0.1–1 μM and GI_50_ values in the mid nanomolar range (Table 1). Notably, both compounds exhibited similar trypanocidal activity to suramin (MIC = 0.1 μM; GI_50_ = 0.035 ± 0.002 μM) (Table 1), one of the drugs used in the treatment of human African trypanosomiasis or sleeping sickness. However, no obvious structure-activity relationship trend could be detected. For example, while the *n*-butyl amide derivative SAL-AM2 was 4.5 times more trypanocidal than salinomycin, the corresponding *n*-butyl ester derivative SAL-E4 was 17 times less trypanocidal than its parent compound (Table 1).

**Table 1:** GI_50_ and MIC values and ratios of salinomycin and monensin derivatives for *T. brucei* and HL-60 cells

	*T. brucei*		HL-60		Selectivity	
Compound	MIC (μM)^a^	GI_50_ (μM)^b^	MIC (μM)^a^	GI_50_ (μM)^b^	MIC ratio^c^	GI_50_ ratio^d^
Salinomycin	1	0.18 ± 0.06	1	0.44 ± 0.21	1	2.4
SAL-E1	10	3.08 ± 0.18	100	35.5 ± 2.4	10	11.4
SAL-E2	10	3.25 ± 0.26	100	34.6 ± 1.9	10	10.6
SAL-E3	10	3.10 ± 0.21	100	33.8 ± 3.5	10	10.9
SAL-E4	10	3.12 ± 0.08	100	32.9 ± 2.5	10	10.5
SAL-E5	10	3.21 ± 0.02	100	38.4 ± 4.2	10	11.0
SAL-E6	10	3.01 ± 0.06	> 100	> 100	> 10	> 33
SAL-E7	0.1–1^e^	0.057 ± 0.029	100	16.4 ± 1.9	100–1000	288
SAL-AM1	10	3.23 ± 0.21	100	38.9 ± 3.2	10	12.0
SAL-AM2	0.1	0.040 ± 0.007	100	14.5 ± 1.3	1000	363
SAL-AM3	10	2.94 ± 0.20	100	7.92 ± 1.95	10	2.7
SAL-AM4	10	2.69 ± 0.51	100	24.5 ± 3.7	10	9.1
SAL-AM5	10	2.69 ± 0.20	100	39.2 ± 1.6	10	14.6
Monensin	0.1	0.029 ± 0.002	10	1.48 ± 0.56	100	51
MON-E1	10	3.06 ± 0.06	100	34.1 ± 2.2	10	11.1
MON-E2	10	2.76 ± 0.10	100	25.3 ± 5.0	10	9.2
MON-E3	10	1.68 ± 0.56	100	20.3 ± 3.1	10	12.1
MON-UR1	1	0.31 ± 0.06	100	23.3 ± 6.6	100	75
Suramin^f^	0.1	0.035 ± 0.002	> 100	> 100	> 1000	> 2857

^a^Data shown are mean values of three independent experiments

^b^Data shown are mean values ± SD of three independent experiments

^c^MIC ratio, MIC_(HL-60)_/MIC_(*T. brucei*)_

^d^GI_50_ ratio, GI_50(HL-60)_/GI_50(*T. brucei*)_

^e^After an incubation period of 72 h, in one of the three experiments a few motile trypanosomes were observed at a concentration of 0.1 μM (but none at 1 μM) while in the two other experiments no motile parasites were found at that concentration. Thus, a range of 0.1–1 μM as MIC value was assigned

^f^Reference control

**Fig. 3:**
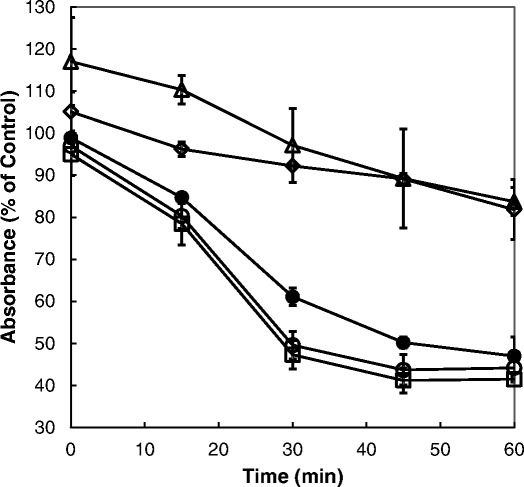
Effect of salinomycin derivatives on the cell volume of bloodstream forms of *T. brucei*. Trypanosomes (5 × 10^7^ cell/ml) were incubated with 100 μM salinomycin (*closed circles*), SAL-E7 (*open circles*), SAL-AM2 (*open squares*), SAL-E4 (*open triangles*), or SAL-AM1 (*open diamonds*) in culture medium. Every 15 min, the absorbance at 490 nm was measured. Mean values ± SD of three experiments are shown. At the time points 30 and 45 min, the values for SAL-E7 and SAL-AM2 were statistically significantly different from the values for salinomycin (*P* < 0.05). At the time points 15, 30, 45 and 60 min, the values for SAL-E4 and SAL-AM1 were statistically significantly different from the values for salinomycin (*P* < 0.05). Note that a decrease in absorbance corresponds to an increase in cell volume

The lack of any clear structure-activity relationship for salinomycin derivatives raised the question whether the two most active substances SAL-E7 and SAL-AM2 share the same mechanism of action as their parent compound. Previously it was shown that the trypanocidal action of salinomycin was due to an influx of Na^+^-ions which subsequently induced swelling of the cells [10]. Change in cell volume can be measured by light scattering at 450–550 nm whereby a decrease in absorbance indicates swelling of cells. In order to be able to record measurable absorbance changes, a high cell density is required. However, since trypanosomes do not survive for a very long time at high cell density in culture, a much higher concentration of ionophores was needed (100 μM) in order to produce fast enough cell swelling [10]. Incubation of trypanosomes with SAL-E7 or SAL-AM2 resulted in cell swelling similar to parasites treated with salinomycin (Fig. 3). However, the derivatives caused a slightly faster swelling of the cells than salinomyicn. After 45 min incubation no further decrease in absorbance was recorded indicating that the endpoint of the swelling process induced by the derivatives was reached. Trypanosomes treated with the parent compound salinomycin continued to swell until the end of experiment. In contrast, when trypanosomes were incubated with derivatives that were found to be less trypanocidal, a prompt increase in absorbance was observed indicating an instantaneous shrinking of the cells (Fig. 3 and data not shown). During further incubation, trypanosomes increased their cell volume but cell swelling was much slower and much less. These results indicate that salinomycin derivatives with modified carboxyl group retain ionophoretic activity but depending on the modification they may transport cations more or less efficient across membranes. However, it seems that the transport efficiency determines the trypanocidal potency of salinomycin derivatives.

For determination of the general cytotoxicity of salinomycin and monensin derivatives, HL-60 cells were used as reference because their sensitivity for approved trypanocides is well established [24, 25]. All derivatives were less cytotoxic towards HL-60 cells than their parent compounds with MIC values of 100 μM and GI_50_ values ranging between 7.9–39 μM (Table 1). Compound SAL-E6 did not affect HL-60 cells, even at 100 μM, indicating that salinomycin 2,4-dinitrobenzyl ester displays no cytotoxicity (Table 1). Overall, the observed cytotoxic activities of the salinomycin derivatives were in good agreement with previously reported findings [11–13].

With the exception of SAL-AM3 (benzyl amide), the selectivity indices (MIC and GI_50_ ratios) of salinomycin derivatives were found to be better than those of the parent compound salinomycin. Most derivatives had selectivity indices of around 10 (Table 1). Compounds SAL-E7 and SAL-AM2 had promising MIC and GI_50_ ratios of > 100 (Table 1). In contrast to salinomycin derivatives, the selectivity indices of most monensin derivatives were found to be inferior to the parent compound monensin (Table 1). Only MON-UR1 (alkyl urethane) had MIC and GI_50_ ratios similar to those of monensin (Table 1). By comparison, drugs used for treatment of African trypanosomiasis have much higher selectivity indices [24, 25]. For example, the reference drug suramin displayed no toxicity towards HL-60 cells with MIC and GI_50_ values greater than 100 μM. Accordingly, the MIC and GI_50_ ratios for suramin were > 1000 and > 2857, respectively (Table 1).

This study has shown that the polyether ionophores can be modified into derivatives with improved trypanocidal and reduced cytotoxic activity. Two salinomycin derivatives, SAL-E7 and Sal-AM2, were identified that, in this respect, were superior to the parent compound. In contrast to salinomycin, derivatization of monensin did not result in compounds with increased trypanocidal activity. One reason for this maybe that monensin itself is already quite trypanocidal (about 6–10 times more active than salinomycin) and, therefore, it might be difficult to improve its trypanocidal activity further by chemical modification.

## Conclusion

Both, SAL-E7 and SAL-AM2, match the activity criteria for drug candidates for African trypanosomiasis (GI_50_ < 1 μM; selectivity > 100) [26]. However, it should be noted that in this study a cancer cell line was used for determining selectivity and that, compared with non-malignant cells, cytotoxicity of both compounds are therefore likely to be overestimated as has been shown for the parent compound salinomycin. For example, the cytotoxicity of salinomyicn in human peripheral blood mononuclear and nasal mucosa cells was determined to be in the mid-micromolar range with 50 % effective concentrations of 30 and 11 μM, respectively [10, 27]. Before developing any salinomycin derivatives into trypanocides, animal experiments should be carried out to establish the *in vivo* activity of the compounds.

## Abbreviations

DMSO, dimethyl sulfoxide; GI_50_, 50 % growth inhibition; MIC, minimum inhibitory concentration; PBS, phosphate-buffered saline
